# Urinary Biomarker Panel to Improve Accuracy in Predicting Prostate Biopsy Result in Chinese Men with PSA 4–10 ng/mL

**DOI:** 10.1155/2017/2512536

**Published:** 2017-02-15

**Authors:** Yongqiang Zhou, Yun Li, Xiangnan Li, Minjun Jiang

**Affiliations:** ^1^Department of Urology, The People's Hospital of Wujiang City, Suzhou, Jiangsu, China; ^2^Department of Urology, Shanghai Shibei Hospital of Jingan District, Shanghai, China; ^3^Department of Anesthesiology, The Third People's Hospital of Yancheng, Yancheng, Jiangsu, China

## Abstract

This study aims to evaluate the effectiveness and clinical performance of a panel of urinary biomarkers to diagnose prostate cancer (PCa) in Chinese men with PSA levels between 4 and 10 ng/mL. A total of 122 patients with PSA levels between 4 and 10 ng/mL who underwent consecutive prostate biopsy at three hospitals in China were recruited. First-catch urine samples were collected after an attentive prostate massage. Urinary mRNA levels were measured by quantitative real-time polymerase chain reaction (qRT-PCR). The predictive accuracy of these biomarkers and prediction models was assessed by the area under the curve (AUC) of the receiver-operating characteristic (ROC) curve. The diagnostic accuracy of PCA3, PSGR, and MALAT-1 was superior to that of PSA. PCA3 performed best, with an AUC of 0.734 (95% CI: 0.641, 0.828) followed by MALAT-1 with an AUC of 0.727 (95% CI: 0.625, 0.829) and PSGR with an AUC of 0.666 (95% CI: 0.575, 0.749). The diagnostic panel with age, prostate volume, % fPSA, PCA3 score, PSGR score, and MALAT-1 score yielded an AUC of 0.857 (95% CI: 0.780, 0.933). At a threshold probability of 20%, 47.2% of unnecessary biopsies may be avoided whereas only 6.2% of PCa cases may be missed. This urinary panel may improve the current diagnostic modality in Chinese men with PSA levels between 4 and 10 ng/mL.

## 1. Introduction

The diagnosis of prostate cancer (PCa) has mostly relied on prostate-specific antigen (PSA) levels and digital rectal examinations (DRE) in the past decades [1]. Nevertheless, the main drawback of PSA is its lack of specificity, resulting in a high negative biopsy rate. In patients with PSA levels between 4 and 10 ng/mL, the negative biopsy rate is as high as 60–70% [2], causing a huge burden for patients and society. Thus, the critical question for improving the diagnosis of PCa should focus on the diagnosis of PCa in patients with PSA levels between 4 and 10 ng/mL. Because it is easy to collect and because prostate cells release directly into the urethra through prostatic ducts after DRE, urine-based tests hold great promise for noninvasive PCa testing. A novel urinary diagnostic biomarker, prostate cancer antigen 3 (PCA3), has been approved by the United States Food and Drug Administration [3]. Recent studies in Japanese [4] and Chinese populations [5] have validated that it is also effective in Asian populations, although these studies showed a relatively lower AUC than some Western studies. Other urinary biomarkers such as metastasis-associated lung adenocarcinoma transcript 1 (MALAT-1), prostate-specific G protein coupled receptor (PSGR), and prostate-specific membrane antigen (PSMA) were confirmed to be associated with PCa as well. Urinary MALAT-1 was associated with the risk of prostate cancer in Chinese populations and may serve as a better biomarker with a higher specificity and AUC than PSA [6]. PSGR overexpression has been shown to be associated with the risk of PCa [7], and urinary PSGR has the potential to be used for the diagnosis of prostate cancer [8]. PSMA is a membrane-bound glycoprotein that is highly restricted to prostatic epithelial cells, and its expression is elevated in prostatic cancer. Previous studies showed that urinary PSMA could be detected and that it was a potential biomarker for the diagnosis of prostate cancer [9].

Nevertheless, the diagnostic accuracy in current clinical practice is still far from satisfactory even with these novel biomarkers, perhaps due to the heterogeneity of PCa itself [10]. The combination of different biomarkers as a panel may help reduce this problem by calculating the overall risk of PCa by considering several biomarkers at once [11]. Recently, novel diagnostic panels based on urinary tests have been developed with improved diagnostic accuracy using previously validated biomarkers [11, 12]. For instance, the most widely studied combination was PCA3 and TMPRSS2-ERG gene fusions. The rationale for this combination was that PCA3 has the highest sensitivity and the TMPRSS2-ERG fusion transcript has the highest specificity for predicting PCa [9]. Nevertheless, the expression level of TMPRSS2-ERG fusion was much lower in Chinese [13], Japanese [14], and Korean [15] populations than in Western populations, indicating that TMPRSS2-ERG fusion may not be applicable to a Chinese population. In addition, there is little information regarding the PSMA and PSGR RNA levels in the urine of Chinese or other Asian populations. This study intends to evaluate the diagnostic efficacy of these biomarkers for the diagnosis of PCa in a Chinese population.

## 2. Methods

### 2.1. Patients and Clinical Specimens

This study was approved by the Institutional Review Board of The People's Hospital of Wujiang City, Shanghai Shibei Hospital, and The Third People's Hospital of Yancheng. The methods were carried out in accordance with approved guidelines. The informed consent of all patients was obtained.

The study was launched and led by The People's Hospital of Wujiang City. All sites shared the same standard operating procedure (SOP) for patient inclusion and sample processing. All the patients were evaluated and recruited in the outpatient department of each site. The inclusion criteria of patients were as follows: (1) men aged 45 years or older with or without family history of prostate cancer; (2) a PSA level between 4 and 10 ng/mL; (3) with or without an abnormal digital rectal examination (DRE); and (4) scheduled for transrectal ultrasound (TRUS) guided systematic prostate biopsy as part of routine medical care. All sites performed a standardized 10–12 core biopsy protocol.

The indications for biopsy were elevated PSA level (4–10 ng/mL) and/or suspicious findings in digital rectal examination (DRE). Patients who had suspicions of urinary tract infections or who received catheterization of the urethra within the previous 2 weeks were excluded. Patients with other known tumors, medical therapy known to affect serum PSA levels, and/or previous treatment for PCa were excluded. Urine samples were collected before prostate biopsy. The prospectively enrolled patients underwent prostate biopsy at The People's Hospital of Wujiang City (70 cases), Shanghai Shibei Hospital (60 cases), and The Third People's Hospital of Yancheng (30 cases).

### 2.2. TRUS Biopsy

Biopsies were performed using an end-fire ultrasound transducer (Falcon 2101; B-K Medical, Inc.) and an automatic 18-gauge needle (Bard, Inc.). In all men, a 10–12-core systematic, laterally directed, TRUS-guided biopsy was performed.

### 2.3. Specimen Collection and Sample Preparation

#### 2.3.1. The Urine Samples Were Collected and the Urine Sediments Were Processed at Each Site

First-catch urine samples were collected following an attentive prostate massage (three strokes for each lobe) before biopsy. The urine samples were cooled immediately on ice and were further processed within 2 hours from collection. Samples were further centrifuged at 2,500 ×g for 15 min at 4°C. The pellets were washed twice with cold PBS (1x). The sediments were then homogenized in TRIzol reagent (Invitrogen: number 15596-026, USA) for RNA extraction and stored at −80°C for further use. The samples were shipped on dry ice and tested at the central laboratory of The People's Hospital of Wujiang City.

### 2.4. Quantitative RT-PCR Analysis

In total, 50 ng of RNA was treated with DNase I (TaKaRa: D2215, TaKaRa, Japan) prior to cDNA synthesis and then amplified using the TransPlex Complete Whole Transcriptome Amplification Kit (WTA2, Sigma-Aldrich, St. Louis, MO, USA). Furthermore, SYBR® Premix Ex Taq™ (Perfect Real Time) (Takara: DRR081A TaKaRa, Japan) was applied in qRT-PCR tests using Applied Biosystems Step One Plus. Cycling conditions were in accordance with the manufacturer's recommendations. The qRT-PCR primers were as follows: PSA-forward primer GTCTGCGGCGGTGTTCTG, PSA-reverse primer TGCCGACCCAGCAAGATC; PCA3 forward primer TGGTGGGAAGGACCTGATGATACAG, PCA3 reverse primer TCTCCCAGGGATCTCTGTGCTTCC; PSMA forward primer GCCCACAGGAACAAGTCCTA, reverse primer CTCTGCAATTCCACGCCTAT; PSGR forward primer CATGGCCTTTGACCGTTATGT, reverse primer GCCAATCTGGGCTGTTACTGTAT; and MALAT1 forward primer CTTCCCTAGGGGATTTCAGG, MALAT1 reverse primer GCCCACAGGAACAAGTCCTA. Briefly, 2 *μ*L of the cDNA solution was amplified using 10 *μ*L SYBR® Premix Ex Taq™ (Perfect Real Time) (2x) (Takara: DRR081A TaKaRa, Japan), 2 *μ*L primers, 0.4 *μ*L ROX Reference Dye (50x), and nuclease-free H_2_O in a final volume of 20 *μ*L. StepOne software version v2.1 (Applied Biosystems, USA) was used for data analysis. A melt-curve analysis was performed at the end of the amplifications. We excluded samples with PSA Ct values of >28 [16] to ensure that a sufficient number of prostate cells were collected. The score for each biomarker (PCA3, PSMA, PSGR, and MALAT-1) was calculated as biomarker mRNA/PSA mRNA × 1000 = 2^Ct(PSA)−Ct(biomarker)^ × 1000. All experiments were performed three times. No amplification of the signal was obtained when nuclease-free water was added instead of cDNA.

### 2.5. Statistical Analysis

Baseline information of the patients and their biomarker scores for positive and negative biopsies were compared using the Mann–Whitney *U*, Student's *t*, Pearson's chi-square, and Fisher's exact tests according to the characteristics of the variables. Univariate and multivariate logistic regression analyses were performed to identify independent predictors of PCa upon biopsy. Variables that were statistically significant in the univariate analysis were further tested in a multivariate analysis. The efficacy of predictors and prediction models was assessed by the area under the curve (AUC) of the receiver-operating characteristic (ROC) curve. Sensitivity, specificity, and positive and negative predicted values were calculated. The differences in AUCs were calculated by *Z*-test. In addition, a decision curve analysis [17] was performed to assess the clinical utility of the base prediction model (with only clinical predictors) and the prediction model with novel biomarkers by quantifying the net benefits with a spectrum of threshold probabilities (10% to 40%, the most relevant range in clinical scenarios). All statistical analyses were performed with MedCalc v.10.4.7.0 (MedCalc Software bvba, Mariakerke, Belgium) and R version 3.1.3 software (R foundation for Statistical Computing, Vienna, Austria, https://www.R-project.org) with the Design and Hmisc Libraries. All *P* values were two-sided. *P* < 0.05 was considered statistically significant.

## 3. Results

### 3.1. Patient Characteristics

**Figure 1:**
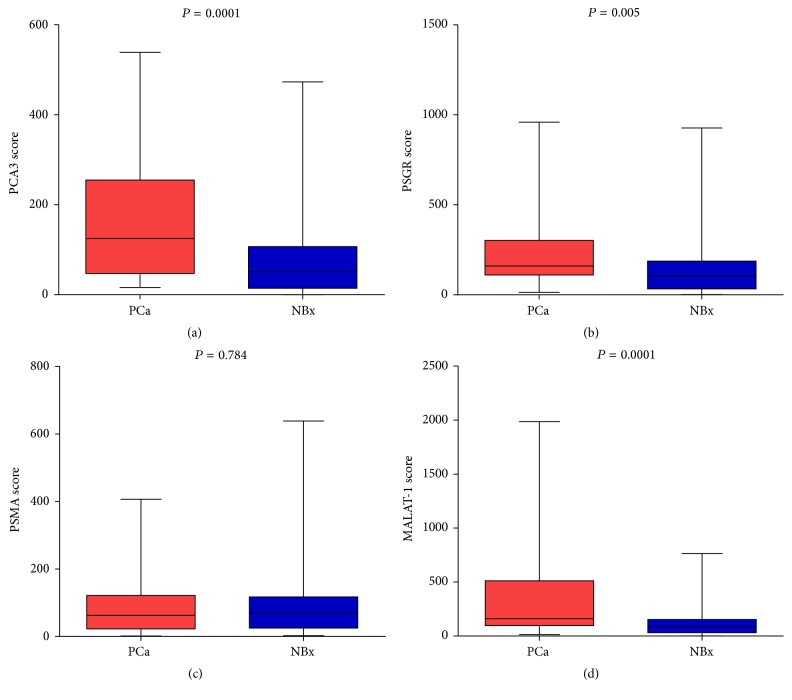
Comparison of PCA3 score (a), PSGR score (b), PSMA score (c), and MALAT-1 score (d) of positive and negative biopsies.* PCa: prostate cancer; NBx: negative biopsy*.

Initially, 160 patients were included in this study. Among them, 14 samples were excluded for insufficient RNA extraction. After quantitative RT-PCR analysis, another 24 patients were excluded for PSA Ct values over 28 [16], indicating an insufficient number of prostate cells collected. Therefore, a total of 122 patients were analyzed. There were 33 cases of patients (27.0%) diagnosed with PCa (Table 1)* and 21 patients (17.2%) diagnosed with high-grade PCa (Gleason Score > 6)*. The mean ages at biopsy were 68.4 and 64.1 years in PCa patients and in patients with negative biopsies, respectively (*P* = 0.006). The mean PSA levels of PCa patients and negative biopsies were 7.0 (SD: 1.7) ng/mL and 7.2 (SD: 1.7) ng/mL (*P* = 0.643), respectively. The PCA3 score, PSGR score, and MALTA-1 score were significantly higher in PCa patients than in patients with negative biopsies (*P* = 0.0001 and 0.0001, resp.; Figure 1). However, the PSMA score was not significantly different in PCa patients and patients with negative biopsies (*P* = 0.784). The DRE was positive in 31 out of the 122 patients (25.4%). Patients with low-grade PCa (Gleason Score < 6) had a lower median MALAT-1 score than those with high-grade PCa (95.1 versus 152.8, *P* = 0.012). The PCA3 score and PSGR score were also higher in high-grade PCa compared with low-grade PCa (116.8 versus 60.0, *P* = 0.005, and 186.5 versus 111.9, *P* = 0.009, resp.), but the PSMA score was similar in high-grade and low-grade PCa (*P* = 0.672).

### 3.2. Univariate Logistic Regression Analyses

Age, prostate volume, % fPSA, DRE results, PCA3 score, PSGR score, and MALAT-1 score were significant predictors for biopsy results in the univariate logistic regression analysis whereas PSA and PSMA were not (Table 2). The PCA3 and MALAT-1 scores represented the most informative parameters in the prediction of PCa (AUC: 0.734 and 0.721, resp.) and were superior to PSA (AUC: 0.525, *P* = 0.0093 and 0.0152, resp.). However, the AUC of PSGR was not significantly higher than that of PSA (*P* = 0.060). The efficacy of these biomarkers in the diagnosis of high-grade PCa is summarized in Table 2. MALAT-1, PCA3, and PSGR were able to differentiate high-grade PCa from negative biopsies and low-grade PCa.

The diagnostic performance of PCa3, PSGR, and MALAT-1 is characterized in Table 3. At a PCA3 cutoff of 23.5, the sensitivity of the PCa3 score was 97.0% and the specificity was 41.6% whereas a cutoff of 214.3 would have a positive predicted value of 50%, indicating that patients with a PCA3 score over this cutoff value would have a high chance of being diagnosed with PCa, especially when taking the PSA range into consideration. The commonly suggested PCA3 cutoff of 35 would have a sensitivity of 81.8% and specificity of 46.1%. The cutoff value for PSGR with the highest Youden index was 93.0, at which the sensitivity and specificity was 81.8% and 49.2%, respectively. For the MALAT-1 score, a cutoff of 109.5 would yield a sensitivity of 72.7% and a specificity of 78.7%. The positive predicted value (+PV), negative predicted value (−PV), positive likelihood ratio (+LR), and negative likelihood ratio (−LR) are listed for these biomarkers. The +PV and −PV for PCA3 at the Youden index point were 38.1 and 97.4, respectively. The −PV and +PV were 40.7 and 85.7, respectively, for MALAT-1 and 37.5 and 88.0, respectively, for PSGR at the Youden index point.

### 3.3. Multivariate Logistic Regression to Evaluate the Diagnostic Performance of Prediction Models

**Figure 2:**
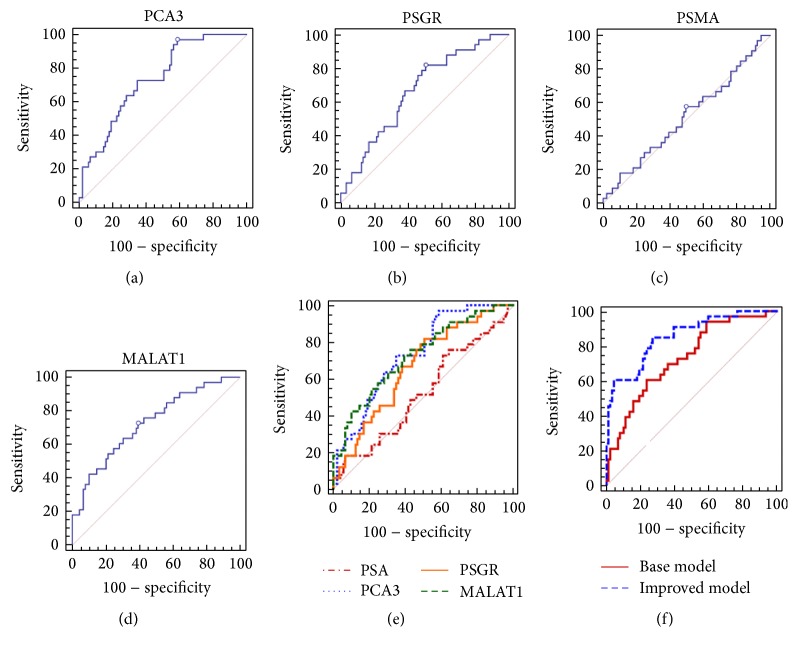
Receiver-operating characteristic curve analysis for evaluating the diagnostic performance of PCA3 score (a), PSGR score (b), PSMA score (c), MALAT-1 score (d), their comparison (e), and the base and improved models (f).

Only three predictors were included in the base prediction model (age, prostate volume, and % fPSA) in multivariate logistic regression analysis with a predicted accuracy of 74.6% and an AUC of 0.733 (95% CI: 0.634, 0.831). However, when we added the three novel biomarkers (PCA3, PSGR, and MALAT-1) to the base model to establish an improved model, we found that the predictive accuracy was improved to 84.4% and the AUC of the improved model was 0.846 (95% CI: 0.766, 0.927) (Table 4, Figure 2).

### 3.4. Decision Curve Analysis for Predicting High-Grade Prostate Cancer

**Figure 3:**
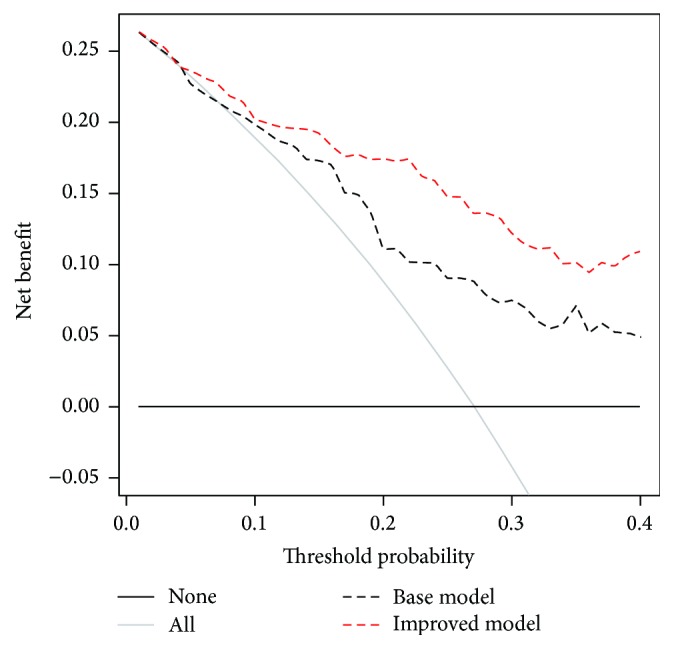
Decision curve analysis for positive biopsy prediction by the base and improved models. The dashed black line indicates the base model; the dashed red line shows the improved model. The horizontal line along the *x*-axis assumes that no patient will have PCa (no patient should undergo a prostate biopsy), whereas the solid grey line assumes that all patients will have PCa (all patients will need to undergo prostate biopsy).

The results of the decision curve analysis indicated that the improved model was superior to the base model in the defined range of clinical interest (10–40%) with a higher net benefit (Figure 3, Table 5). It is clear that both models outperformed the strategy of performing biopsies on every patient (“treat all”). The numbers of PCa and high-grade PCa cases that were missed and the reductions in unnecessary biopsies according to each threshold probability for the two models are summarized (Table 6). For instance, if a probability threshold of 20% was used, although 2 PCa patients (6.2%) would be missed using the improved model (including 1 high-grade PCa) which is more than the 1 patient that would be missed when using the base model, the improved model would save 42 (47.2%) patients from getting unnecessary biopsies (versus 11 patients in the base model). At a probability of 35% or 40%, the improved model would reduce the number of unnecessary biopsies while missing fewer PCa patients.

## 4. Discussion

To the best of our knowledge, this is the first study investigating the diagnostic performance of PCA3, PSGR, MALAT-1, and PSMA in an Asian population. We have validated that PCA3, PSGR, and MALAT-1 scores were able to discriminate PCa patients from patients with negative biopsies. Further analyses indicated that the prediction model incorporating these three biomarkers improves the diagnostic accuracy compared with the current clinical modality. The decision curve analysis illustrated that this prediction model would greatly benefit patients undergoing prostate biopsy by reducing the number of unnecessary biopsies.

There is some strength in this study. First, this study presented the first evaluation of these four biomarkers in predicting PCa in Chinese men with PSA levels between 4 and 10 ng/mL. The AUC of the PCA3 score is the highest, followed by MALTA-1 and PSGR. Second, although the diagnostic performance of biomarker panels similar to those in this study was previously validated in Western populations, this study is the first that was conducted in Asians that demonstrated an improved diagnostic performance (AUC = 0.846). Third, this study tested whether novel biomarker panels would significantly improve the diagnosis of PCa; we found that MALAT-1 has similar discriminative power to that of PCA3, which validated our previous study [6]. Fourth, we believe that the design of this study is guaranteed by the fact that these biomarkers have been previously confirmed to be associated with PCa, and combining these biomarkers is in accordance with the ethical considerations of clinical trials in the future.

The diagnosis of PCa in patients with PSA levels between 4 and 10 ng/mL is quite challenging because patients with PSA levels over 10 ng/mL have a much higher risk of PCa; however, it is rather difficult to differentiate cases with PCa from those without PCa in men with PSA levels between 4 and 10 ng/mL, the so-called “PSA grey zone.” The combined performance of this urinary biomarker panel is relatively high in patients with PSA levels between 4 and 10 ng/mL, especially in clinical practice when considering the threshold probability of triggering a prostate biopsy. As reported above, if a doctor biopsied a man only if his probability of PCa was over 20%, this improved model would save almost half (47.2%) of patients from unnecessary biopsies, at the cost of missing only 2 PCa patients (including 1 high-grade PCa case). In addition, these urinary biomarkers could be measured simultaneously with only one urine sample, which makes this method highly cost-effective. We consider this improvement of clinical relevance and such evidence is supported by the application of this panel in Chinese men.

Inconsistent with previous studies in Western populations, PSMA was not a significant predictor of the biopsy results in this study. PSMA protein was shown to be upregulated in PCa tissue compared with benign prostate tissues [18] and was able to differentiate PCa and non-PCa with a special protein-based detection method [19]. The expression of PSMA RNA in urine was closely related to its protein expression in tissue [18]. This induction is quite reasonable, as studies have indicated that the RT-PCR based PSMA level in urine was superior to PCA3 in predicting cancer. At 70% specificity, the sensitivities for PSMA, PCA3, and PSGR in predicting PCa were 64%, 46%, and 61%, respectively [20]. We speculated multiple reasons for this disparity. First, the previous study was performed with a limited number of patients (82 cases with PSA 4–10 ng/mL) [20]. Second, it may be due to the instability of the method of this urinary test. However, we failed to identify any differences in performing the experiments with PSMA and other biomarkers in this study. Third, it is possible that the efficacy of RT-PCR based PSMA tests was confined to the Asian population compared with Western populations due to racial disparities. However, because there are no relevant reports, such differences should be further investigated. We emphasize that urinary RT-PCR-based PSMA tests merit further investigation, but we believe that PSMA has the potential to be a robust biomarker. Recently, a number of studies confirmed that PSMA serves as a good target for molecular imaging in PET examinations [21, 22] and potential targets in the treatment of metastatic castration-resistant prostate cancer (mCRPC) [23]. However, some limitations of this study must be acknowledged. First, the sample size is limited. Second, we failed to find the exact reason for the disparity in the diagnostic performance of PSMA between this study and studies in Western populations.

In conclusion, we demonstrated that urinary RT-PCR based PCA3, PSGR, and MALAT-1 scores and panels of these biomarkers in combination could serve as a noninvasive method for detecting PCa in Chinese men with PSA levels between 4 and 10 ng/mL. Applying a probability threshold of 20%, the improved model would avoid almost half of unnecessary biopsies while only missing 6.2% of PCa cases. Future large-scale studies are needed to confirm the efficacy of this panel in the diagnosis of prostate cancer.

## Figures and Tables

**Table 1:** Clinical characteristic of men with positive and negative biopsy.

Median (IQR)	All patients		*P* value
	Prostate cancer	Negative biopsy	
No. pts	33	89	
Age, mean (SD), years	68.4 (6.7)	64.1 (7.7)	*P* ^#^ = 0.006
PSA, mean (SD), ng/mL	7.0 (1.7)	7.2 (1.7)	*P* ^#^ = 0.643
Prostate volume, mL	51.6 (38.2, 69.7)	39.9 (28.0, 55.9)	*P* ^*∗*^ = 0.008
% fPSA, %	15.0 (10.6, 18.5)	17.0 (13.3 to 22.7)	*P* ^*∗*^ = 0.048
PCA3	125.0 (48.4, 252.7)	51.5 (14.5 to 104.7)	*P* ^*∗*^ = 0.0001
PSGR	160.3 (111.5, 298.9)	105.8 (32.9 to 187.0)	*P* ^*∗*^ = 0.005
PSMA	62.7 (22.5, 120.9)	69.2 (25.4, 116.0)	*P* ^*∗*^ = 0.784
MALTA-1	160.3 (101.4 to 499.5)	85.5 (31.1 to 151.8)	*P* ^*∗*^ = 0.0001
Positive DRE	11/33	20/89	*P* ^*∗∗*^ < 0.01

IQR: interquartile range; no. pts: number of patients; PSA: prostate-specific antigen; % fPSA: percent free PSA; PCA3: prostate cancer antigen 3; PSGR: prostate-specific G protein coupled receptor; PSMA: prostate-specific membrane antigen; MALAT-1: metastasis-associated lung adenocarcinoma transcript 1; *P*^*∗*^: Mann–Whitney *U* test; *P*^#^: independent sample test; *P*^*∗∗*^: chi-square test.

**Table 2:** Univariate logistic regression analyses of predictors for predicting prostate cancer.

Variables	OR (95% CI); *P*	AUC (95% CI)
Age	1.089 (1.0227, 1.160); 0.004	0.668 (0.577, 0.750)
PSA	0.945 (0.747, 1.197); 0.640	0.525 (0.408, 0.642)
Prostate volume	0.975 (0.954, 0.996); 0.020	0.657 (0.566, 0.741)
% fPSA	0.0003 (0, 0.241); 0.018	0.617 (0.524, 0.703)
DRE	1.725 (0.717, 4.152); 0.224	0.554 (0.437, 0.672)
PCA3	1.006 (1.002, 1.010); 0.001	0.734 (0.641, 0.828)
PGSR	1.002 (1.000, 1.004); 0.026	0.666 (0.575, 0.749)
PSMA	0.999 (0.995, 1.003); 0.621	0.516 (0.398, 0.634)
MALAT-1	1.003 (1.001, 1.005); 0.002	0.727 (0.625, 0.829)

PSA: prostate-specific antigen; % fPSA: percent free PSA; DRE: positive digital rectal examination results; PCA3: prostate cancer antigen 3; PSGR: prostate-specific G protein coupled receptor; PSMA: prostate-specific membrane antigen; MALAT-1: metastasis-associated lung adenocarcinoma transcript 1.

**Table 3:** Sensitivity and specificity of three biomarkers with efficacy in predicting prostate cancer in patients with PSA 4–10 ng/mL.

—	Criterion	Sensitivity	95% CI	Specificity	95% CI	+LR	−LR	+PV	−PV
PCA3	23.5^*∗*^	97.0	84.2–99.9	41.6	31.2–52.5	1.7	0.1	38.1	97.4
	30.4	90.9	75.7–98.1	43.8	33.3–54.7	1.6	0.2	37.5	92.9
	35.4	81.8	64.5–93.0	46.1	35.4–57.0	1.5	0.4	36.0	87.2
	214.3	27.3	13.3–45.5	89.9	81.7–95.3	2.7	0.8	50.0	76.9

PSGR	24.2	90.9	75.7–98.1	20.2	12.4–30.1	1.1	0.5	29.7	85.7
	93.0^*∗*^	81.8	64.5–93.0	49.4	38.7–60.2	1.6	0.4	37.5	88.0
	623.3	12.1	3.4–28.2	96.6	90.5–99.3	3.6	0.9	57.1	74.8

MALAT-1	32.8	90.9	75.7–98.1	25.8	17.1–36.2	1.2	0.4	31.3	88.5
	109.6^*∗*^	72.7	54.5–86.7	60.7	49.7–70.9	1.9	0.5	40.7	85.7
	156.5	54.6	36.4–71.9	78.7	68.7–86.6	2.6	0.6	48.6	82.4

PSA	9.6	90.9	75.7–98.1	11.2	5.5–19.7	1.02	0.81	27.5	76.9
	7.95^*∗*^	72.7	54.5–86.7	39.3	29.1–50.3	1.20	0.69	30.8	79.5
	5.1	18.18	7.0–35.5	91.0	83.1–96.0	2.02	0.90	42.9	75.0

% fPSA	21.9	90.9	75.7–98.1	28.1	19.1–38.6	1.26	0.32	31.9	89.3
	23.2	100.0	89.4-100.0	24.7	16.2–35.0	1.33	0.00	33.0	100.0
	9.1	21.2	9.0–38.9	89.9	81.7–95.3	2.10	0.88	43.7	75.5

^*∗*^Indicating the cutoff value of the Youden index. PA: predictive accuracy; +LR: positive likelihood ratio; −LR: negative likelihood ratio; +PV: positive predicted value; −PV: negative predicted value.

**Table 4:** Multivariate logistic regression analyses of the base model and the improved model for predicting prostate cancer.

Variables	Base model^‡^	Improved model
	OR (95% CI); *P*	OR (95% CI); *P*
Age	1.080 (1.010, 1.155); 0.024	1.058 (0.978, 1.144); 0.159
Prostate volume	0.979 (0.958, 1.001); 0.063	0.984 (0.960, 1.009); 0.221
% fPSA	0.001 (0.00001, 1.438): 0.063	0.00001 (0, 0.277); 0.024
PCA3	—	1.008 (1.003, 1.012); 0.002
PGSR	—	1.002 (1.000, 1.005); 0.036
MALAT-1	—	1.004 (1.001, 1.006); 0.003
PA (%)	74.6%	84.4%
Increment PA (%)	—	9.8%
AUC (95% CI)	0.733 (0.634, 0.831)	0.857 (0.780, 0.933)
Increment AUC (95% CI)	—	0.124

PCA3: prostate cancer antigen 3; PSGR: prostate-specific G protein coupled receptor; MALAT-1: metastasis-associated lung adenocarcinoma transcript 1; AUC: area under the curve; 95% CI: 95% confidential interval.

**Table 5:** Net benefit and reduction in avoidable biopsies in predicting high-grade prostate cancer for the base model and improved model compared to the ‘‘treat-all” strategy to biopsy every patient for different threshold probabilities in the same range.

Threshold probability (%)		15	20	25	30	35	40
Net benefit	Base model	17.3	11.1	9.0	7.5	7.1	4.9
	Improved model	19.2	17.4	14.8	12.2	10.2	10.9
	Treat all	14.2	8.8	2.7	−4.2	−12.2	−21.6

Net reduction in the number of biopsies	Base model	17.8	9.0	18.9	27.3	35.9	39.8
	Improved model	28.7	34.4	36.1	38.3	41.6	48.8

**Table 6:** Number of high-grade prostate cancers missed and reduction in biopsies according to threshold probability in the range of 10–40% for the base model and improved model.

Probability cutoff, %	Model	PCa missed, number (%)	High-grade PCa missed, number (%)	Unnecessary biopsies spared, number (%)
15	Base model	1 (3.0%)	0 (0%)	22 (24.4%)
	Improved model	1 (3.0%)	1 (3.0%)	35 (39.3%)
20	Base model	1 (3.0%)	0 (0%)	11 (12.3%)
	Improved model	2 (6.1%)	1 (3.0%)	42 (47.2%)
25	Base model	1 (3.0%)	0 (0%)	23 (25.9%)
	Improved model	3 (9.1%)	2 (6.1%)	44 (49.5%)
30	Base model	2 (6.1%)	1 (3.0%)	33 (37.4%)
	Improved model	3 (9.1%)	2 (6.1%)	47 (52.5%)
35	Base model	4 (12.1%)	3 (9.1%)	44 (49.2%)
	Improved model	3 (9.1%)	2 (6.1%)	51 (57.0%)
40	Base model	7 (21.2%)	5 (15.2%)	49 (54.6%)
	Improved model	5 (15.2%)	4 (12.1%)	60 (66.9%)
